# An investigation on the role of oxytocin in chronic neuropathic pain in a Wistar rat model

**DOI:** 10.1016/j.ynpai.2024.100152

**Published:** 2024-03-07

**Authors:** Michaela de Kock, Sean Chetty, Ahmed Sherif Isa, Lihle Qulu-Appiah

**Affiliations:** a Division of Medical Physiology, Faculty of Medicine and Health Science, Stellenbosch University, South Africa; b Department of Human Physiology, Ahmadu Bello University, Nigeria; c Anaesthesiology and Critical Care, Faculty of Medicine and Health Science, Stellenbosch University, South Africa

**Keywords:** Chemotherapy-induced peripheral neuropathy, Chronic pain, Oxytocin, Analgesia, Anxiolytic

## Abstract

***Introduction*** Chemotherapy-induced peripheral neuropathy (CIPN) is a dose-limiting side effect with ineffective preventative and curative treatment. Currently, only Duloxetine has been recommended as effective treatment for CIPN, which has shown individual-dependent, short-term analgesic effects, with limiting adverse effects and poor bioavailability. The neuropeptide, oxytocin, may offer significant analgesic and anxiolytic potential, as it exerts central and peripheral attenuating effects on nociception. However, it is unknown whether the intervention administered in a model of CIPN is an effective therapeutic alternative or adjuvant. ***Materials and Methods*** The intervention was divided into two phases. Phase 1 aimed to induce CIPN in adult Wistar rats using the chemotherapeutic agent Paclitaxel. Mechanical (electronic von Frey filament) and thermal (acetone evaporation test and Hargreaves test) hypersensitivity testing were used to evaluate changes due to the neuropathic induction. Phase 2 consisted of a 14-day intervention period with saline (o.g.), duloextine (o.g.), or oxytocin (i.n.) administered as treatment. Following the intervention, anxiety-like behaviour was assessed using the elevated plus maze (EPM) and light–dark box protocols. Analysis of peripheral plasma corticosterone, peripheral plasma oxytocin, and hypothalamic oxytocin concentrations were assessed using ELISA assays. ***Results*** The findings showed that we were able to successfully establish a model of chemotherapy-induced peripheral neuropathy during Phase 1, determined by the increase in mechanical and thermal nociceptive responses following Paclitaxel administration. Furthermore, the animals treated with oxytocin displayed a significant improvement in mechanical sensitivity over the intervention phase, indicative of an improvement in nociceptive sensitivity in the presence of neuropathic pain. Animals that received Paclitaxel and treated with oxytocin also displayed significantly greater explorative behaviour during the EPM, indicative of a reduced presence of anxiety-like behaviour. ***Conclusion*** Our results support the hypothesis that intranasally administered oxytocin may augment the analgesic and anxiolytic effects of duloxetine in a chemotherapy induced peripheral neuropathy model in a Wistar rat. Future studies should consider administering the treatments in combination to observe the potential synergistic effects.

## Highlights

• An investigation of oxytocin as a potential alleviator or adjuvant treatment for chemotherapy-induced peripheral neuropathy (CIPN) is performed using a well-known animal model.
• Behavioural assessment is used to monitor the progression of the neuropathy, followed by neurochemical assessment of oxytocin and corticosterone.
• Animals treated with oxytocin displayed a significant improvement in mechanical sensitivity over the intervention phase, as well as greater displays of explorative behaviour in comparison to their counterparts.
• Intranasally administered oxytocin may augment the analgesic and anxiolytic effects of duloxetine in a CIPN model.

## Introduction

Chronic pain, defined as persisting pain for more than 3 consecutive months, is one of the leading causes of human disability and suffering (Treede, 2019). One of the most prevalent forms of chronic pain is neuropathic pain (NPP), defined as, “pain arising as a direct consequence of a disease or lesion of the somatosensory nervous system at either the central or peripheral level” (Treede, 2019). With a global estimated 6.9–10 % of adults experiencing pain with neuropathic characteristics (Murnion, 2018, Tai et al., 2018, van Hecke et al., 2014), it is a crisis that requires drastic action to improve current treatment outcomes. This high prevalence is not only a representation of human suffering, but also of debilitating social and financial burden the individual, their family, and the healthcare system are confronted with (Dahlhamer, 2018). Various factors are associated with the high prevalence of chronic NPP, including infection, prolonged/chronic stress, nerve trauma/entrapment, excessive alcohol consumption, stroke, post-amputation, chronic inflammation, diabetes, HIV, surgical procedures, and cancer (Patel et al., 2018, Tai et al., 2018, Treede, et al., 2019, Yan et al., 2017). Cancer itself is not necessarily the risk factor, but rather the extensive treatment thereof.

Chemotherapy-induced peripheral neuropathy (CIPN) is a frequently encountered complication arising from the treatment of cancer using conventional antineoplastic agents (Colvin, 2019). This condition can progress to become a dose-limiting side effect (Molassiotis, 2019) with ineffective preventative and curative treatment (Seretny, 2014) due to the condition’s complexity. During chemotherapy, 70.8 % of patients reported CIPN symptoms (Bao, 2016), and 30 % of patients presented with chronic CIPN at a 6- month follow-up post-chemotherapy treatment (Seretny, 2014). Currently, only duloxetine has been recommended as an effective treatment for CIPN (Colvin, 2019). However, duloxetine has only shown individual-dependent, short-term analgesic effects (di Cesare Mannelli, 2017), with limiting adverse effects and poor bioavailability (Chahal, Sodhi and Madan, 2020). Due to extensive central involvement (Elman & Borsook, 2016) and a plethora of associated risk factors (Li, 2017), less than half of patients treated with conventional analgesics experiences pain relief (Muñoz, 2018), and usually require the reduction or cessation of chemotherapy treatment (Niemand et al., 2020). CIPN is associated with a pro-inflammatory state and upregulation of anxiety, depression, and sleep disturbances (Bao, 2016), contributing to a decline in physical independence and significant medical costs (Molassiotis, 2019).

The presence of chronic pain is considered to be a self-amplifying stressor, perpetuating the dysfunctional stress response and subsequent accumulation of glucocorticoids, primarily cortisol, within the physiological milieu (Elman & Borsook, 2016). Chronic disruptions due to persistent stressors increase the risk of developing psychiatric disorders known to be associated with neuropathic pain, such as anxiety and depression (Caudle, 2016). These disorders often occur as a result of altered hypothalamic–pituitary–adrenal (HPA) axis function (Fischer et al., 2017). HPA axis dysfunction is associated with chronic elevated levels of cortisol (Tang et al., 2019) and a reduced ability to cope with stressful events (Godoy, 2018). Subsequently, the patient experiences a prolonged deterioration in quality of life, for which effective treatment is minimal (Seretny, 2014).

To develop effective, long-term treatment modalities, HPA axis activity attenuation is crucial. The use of the neuropeptide, oxytocin, as an analgesic and anxiolytic has gained significant traction in recent literature (Boll, 2018). Known to be involved in the modulation of social behaviour and downregulation of the stress response and anxiety (Gao, et al., 2015, Neumann, 2007), oxytocin exerts central and peripheral attenuating effects on nociception (Tracy, 2015), thus providing the efficacy required to treat neuropathic pain. To our knowledge, there has only been one investigation into the nociceptive efficacy of chronically administered oxytocin in an animal model. Gonzalez-Hernandez *et al* (2019) demonstrated the analgesic potential using a spinal nerve ligation model. However, the therapeutic potential of this neuropeptide has not been investigated in an animal model of CIPN. Therefore, our study aimed to firstly establish a reputable model of CIPN, and thereafter evaluate any ameliorative effects facilitated by oxytocin. As oxytocin offers the most effective analgesic benefits through central pathways and is inefficient when passing the blood brain barrier (BBB) (Rash, Aguirre-Camacho and Campbell, 2014), our study utilised a method of intranasal administration to bypass the BBB (Tracy, 2015) with the aim to optimise the analgesic and anxiolytic effects.

## Methods and Materials

**Animals** A total of 61 male (M) and female (F) Wistar rats (M = 30; F = 31) were used in this study design, which was in accordance with conditions approved by Stellenbosch University Research Ethics Committee: Animal Care and Use (ACU-2021–22038). Animals were received from the animal unit at 3 weeks of age and placed in groups of 5 per cage, separated by sex. Once animals were separated into respective cages, the littermate remained constant throughout the entirety of the study. All animals were housed in standard environmental conditions, which consisted of a climate-controlled room with a temperature set at 20 ± 1 °C, a relative humidity of 50 ± 10 %, a constant 12-hour light/dark cycle (lights on at 06:00, off at 18:00), food and water *ad libitum*, dust-free wood shavings, and environmental enrichment consisting of a red rat tunnel. Cages were cleaned at minimum once weekly, and more frequently as the animals grew. All experimental pain procedures were conducted in strict accordance with the IASP ethical guidelines (Zimmerman, 1986) and performed at the same time of day (between 7:00 and 15:00) to avoid diurnal variations in animal responses (Zang, 2019). Animals were left undisturbed until the age of 3 months.

**Experimental Blinding** Due to the nature of the experiments with regards to administration methods (intranasally, orally, and intraperitoneally), as well as the necessity to reduce the number of researchers working with the animals, the researcher was not blinded to groupings throughout the study. Care was taken to reduce researcher bias as much as possible, and randomization was introduced wherever possible.

**Experimental Procedure (Phase 1)** The study was divided into two phases. During Phase 1, animals were randomly separated into two groups defined as Saline (SAL, n = 30, F = 15) and Paclitaxel (PAC, n = 31, F = 16). Phase 1 took place over a 3-week period. The first week consisted of various baseline behavioural assessments, followed by week 2 where neuropathic pain was induced using the chemotherapeutic agent; Paclitaxel. Phase 1 concluded with a monitoring period of 8 days from the final injection to the start of the intervention (week 3). During this monitoring week, the animals were only disturbed during cage cleaning. This monitoring week consisted of twice-daily observations, where any unusual behaviour was noted. The veterinary technician performed routine observations in addition to the twice-daily observations, to ensure no abnormal behaviour was overlooked. This monitoring duration was selected based on previous literature demonstrating the effects of Paclitaxel when administered following this cycle. Animals that received the same dosage in previous studies demonstrated heightened sensitivity 7 days following final injection, with a peak between 21 and 28 days (Dugget *et al,* 2016).

**Behavioural Assessment: Analgesic Testing** Following maturation and habituation, all animals underwent mechanical and thermal hypersensitivity testing on the same day to obtain baseline readings. Acclimatisation in the experimental room began at 6:00 for all behavioural testing throughout the intervention period. During all testing, animals were acclimated to the transparent compartments for 30–60 min, until they were alert and weight bearing on all four paws. Following all behavioural tests, animals were returned to their home cages, and all apparatus was thoroughly wiped with 70 % ethanol, to prevent the transfer of stress between animals via olfactory stimulation. During Phase 1, the following analgesic tests were used:

***Mechanical Allodynia*** A calibrated electronic von Frey filament was used to assess mechanical tactile hypersensitivity to an innocuous stimulus (i.e., allodynia). The principle of the assessment is to evaluate the presence or extent of aversive behaviour in response to a mechanical stimulus (Deuis et al., 2017). Three days prior to the official testing, habituation training commenced. Each day, animals were randomly placed in elevated, individual, transparent Perspex compartments resting on a mesh wire at 7:00. Animals were removed from the compartments at the end of a 60-minute period (González-Hernández, 2019) and returned to their home cages. The transparent compartments were thoroughly wiped with 70 % ethanol between every animal.

On the day of testing, animals were randomly assigned to a compartment and an acclimatisation period of 30–60 min was allowed, until alert and weight bearing on all four paws. The testing procedure then began between 7:30 – 8:00. A single, rigid filament was applied perpendicularly to the plantar portion of the right hind paw with gradual increasing force until a paw withdrawal response was elicited (Deuis et al., 2017). The force (in grams) required to evoke the response was automatically recorded by the apparatus (Electronic von Frey Anesthesiometer; IITC Life Science), as well as manually recorded by the researcher. All animals were tested on the right paw before returning to the first animals’ left paw, to allow for a minimum of 5 min to elapse (Tai et al., 2018). Any hindpaw movements deemed as a result of reflex/locomotion were disregarded and the test trial was repeated during the following round of testing (Tai et al., 2018). Baseline measures were averaged to represent the paw withdrawal threshold (PWT).

***Cold Allodynia*** An acetone evaporation test was used to evaluate cold allodynia. The principle of the assessment is to measure aversive behaviours evoked by evaporative cooling (Deuis et al., 2017). This assessment was performed following mechanical allodynia testing, between 10:00 and 12:00. The same transparent compartments resting on wire mesh were used. Once the animals were acclimated, the test commenced. A drop of acetone (100 µL) was gently applied to the plantar surface of the left hind paw of the animal using a 1 mL syringe, with care taken to avoid spreading the acetone onto the animals’ fur (Deuis et al., 2017). Care was also taken to ensure the tip of the syringe did not come into contact with the animals’ hind paw. Once applied, a stopwatch was started and the total frequency of responses was graded according to the criteria noted (shaking/sudden withdrawing of paw, biting/licking of paw, cleaning of paw). Frequency was recorded manually over a 1-minute observation period. All animals within a group were tested on the left paw before returning to test the first animal, ensuring a minimum of 10 min elapsed before the next acetone application to the same hind paw (Gong et al., 2018, Vachon et al., 2013).

***Thermal Hyperalgesia*** The Hargreaves test was used to evaluate heat hypersensitivity to a noxious stimulus (i.e. hyperalgesia). The principle of the assessment is to quantify the heat threshold of the animals’ hind paw upon application of a radiant heat stimulus (Deuis et al., 2017). Animals were given a 2-hour respite between 12:00 and 14:00 following the acetone evaporation test (Grégoire, 2020). Thereafter, the assessment took place between 14:00 and 15:00. The animal was placed in the transparent compartment on top of a glass bottom surface and left to acclimatise (González Hernández *et al*., 2019). A radiant heat source (Plantar Test Analgesia Meter, IITC Life Science) was positioned underneath the mid-plantar surface of the right hind paw. The time taken for a sharp withdrawal from the heat stimulus to occur was recorded and termed the paw withdrawal latency (Deuis et al., 2017). The intensity of the light source was set at 30 % (Ibrahim & Ehrlich, 2020) and a pre-determined cut-off time of 20 s was set, to prevent tissue damage (Deuis et al., 2017). All animals within a group were tested on the right hind paw prior to returning to test the first animal, ensuring a minimum of 10 min had elapsed before the next heat application to the same hind paw (González-Hernández, 2019).

**Neuropathic Pain Induction** A week following the analgesic behavioural testing, animals were administered the chemotherapeutic agent Paclitaxel, used to generate a model of chemotherapy-induced neuropathic pain (CIPN). The first day of administration marked the start of the experimental procedure, and was denoted as Experimental Day 1 (ED 1). We selected a dose concentration and administration procedure frequently used in literature (Soo Shim, *et al*. 2019; Duggett, 2016; Xu, *et al*. 2016; Griffiths, & Flatters,. 2015). 6 mg/ml Paclitaxel solution was diluted with saline solution (0.9 % sodium chloride) until a concentration of 2 mg/ml was obtained. The antineoplastic drug was then administered via intraperitoneal (*i.p.*) injections on alternating days (ED 1, 3, 5, 7) to mimic the conventional chemotherapy cycles (Duggett, 2016). On the days of injection, animals were transported to the experimental room at 6:00 and allowed to acclimate for 1 h. During the acclimation period, the researcher prepared the Paclitaxel solution. At 7:00, the *i.p*. injection was administered (2 mg/kg fresh Paclitaxel solution per injection, a total dosage of 8 mg/kg), and the animal was immediately placed into a temporary cage separate from their littermates. Once all animals had received injections, they were transferred back to their home cages and monitored for a 30-minute period, to ensure any adverse effects were immediately attended to. Thereafter, animals were returned to their home room and left undisturbed until the next injection. Animals were observed twice daily to assess ongoing symptoms over the induction period. No animals were lost during this phase. The groups that received the vehicle solution underwent the same procedure. An equal concentration of saline solution was administered intraperitoneally. Once all animals had undergone the final day of injections, they were returned to their home cages and left undisturbed for a 1-week monitoring period (ED 8–13). Animals were observed twice daily using individual welfare monitoring sheets **(Addendum 1)** to ensure any excessive pain symptoms were identified. To assess whether neuropathic pain was induced, the analgesic behavioural assessments were repeated seven days following the final injection, denoted as ED 14.

**Experimental Procedure (*Phase 2)*** Phase 2 consisted of a 14-day treatment period commencing on ED 15, and included the following treatments: saline solution (CTRL), Duloxetine (DUL), and oxytocin (OXY). The groups from Phase 1 were further separated into six groups using computer-generated randomization, to produce the following groups: control (n = 10, M = 5, F = 5), Paclitaxel (n = 10, M = 5, F = 5), Duloxetine (n = 10, M = 5, F = 5), Oxytocin (n = 10, M = 5, F = 5), Paclitaxel-duloxetine (n = 10, M = 5, F = 5), Paclitaxel-oxytocin (n = 11, M = 5, F = 6). During the 14-day period, the analgesic behavioural assessments were conducted on a weekly basis (twice over the intervention period) to continue monitoring for any changes in mechanical and thermal sensitivity. At the end of the intervention period, anxiolytic behavioural assessments were conducted, followed by euthanasia 24–48 h later.

**Treatment** During the treatment period, the respective treatment was administered daily. Animals were transferred to the experimental room at 6:00 and allowed to acclimate for 1 h. During the acclimatisation hour, the drug solutions were prepared by the researcher. All treatment administration took place from 7:00 onwards. Testing took place a minimum of 1 h following drug administration, to ensure adequate time for restoration to baseline stress levels. Immediately following the administration, the animal was placed into a temporary cage separate from their littermates. Once all animals had received treatment, they were transferred back to their home cages and monitored for a 30-minute period, to ensure any adverse effects were not overlooked. Following this monitoring period, animals were either returned to their home room or remained in the experimental room, if they were scheduled for analgesic behavioural assessment**.** Animals allocated to the control groups were administered saline solution via oral gavage over the treatment period (1 mL/kg). Duloxetine Hydrochloride powder (Sigma-Aldrich, CAS # 136434–34-9) was diluted in saline and administered via oral gavage (30 mg/kg). The use of duloxetine as treatment for CIPN in animal models has been well-established. We based our dose on several of these interventions which demonstrated favourable results, being an alleviation of nociceptive behaviour (Selvy, *et al.* 2022; Staurengo-Ferrari et al., 2022, Chahal et al., 2020, Meng et al., 2019, Zhang et al., 2018). Finally, oxytocin (Sigma-Aldrich, O4375, CAS # 50–56-6) was diluted in saline and administered intranasally (2 x 10 uL into each nostril) (Neumann *et al*., 2013). As this is a novel model of CIPN, there have been no prior studies indicating an optimal dose of oxytocin in this specific model. We therefore based our dosage on previous studies focused on chronic pain management using intranasal oxytocin (Wang, *et al.* 2021; Yang, *et al.* 2019; Neumann *et al*., 2013). We also took into consideration that the normal oxytocin concentration within the cerebral spinal fluid of rats is 0.5–1 ug/ml, and that only a finite amount of oxytocin administered would likely reach the central nervous system.

**Behavioural Assessment: Anxiolytic Testing** Following the intervention period, all animals underwent anxiety-like behavioural assessments 24 h following the completion of the final analgesic behavioural testing protocol. Both protocols were recorded using a GoPro Hero 7 positioned directly above the apparatus. Analysis of behaviour was performed retrospectively using Behavioural Observation Research Interactive Software (BORIS), Version 7.13. Acclimatisation in the experimental room began at 6:00, to ensure adequate time for the animals to assume a relaxed state for the assessments. The respective behavioural test of that day commenced at 7:00. Following the completion of each test, the animal was transferred to a new cage until all cage mates had undergone the protocol. The entire group was then returned to their home cage and monitored for 30 min before being returned to their home room. The apparatus was thoroughly wiped with 70 % ethanol between each test. The following tests were used:

***Light/dark Box*** This assessment is separated into three sessions, two training sessions and a testing session. Each session transpired 24 h following the last. The principle of the assessment is to evaluate the conflict between the animal’s exploratory drive versus its aversion to bright, open spaces. Anxious animals tend to avoid the illuminated compartment (Abboud, 2021), therefore the percentage of time spent in the dark compartment is used as an indication of anxiety. Exploratory behaviour can be represented by the percentage of time spent in the illuminated compartment. The test session followed the same protocol as described below; except the light/dark box (LDB) was divided by a removable partition with a small aperture to allow for free access between compartments. This introduced a novel obstacle to the protocol and can be used to evaluate the animals learning and memory, by analysing the latency to navigate the dark compartment.

On the respective assessment days, the box was placed on a marked area on the experimental room floor with the GoPro positioned directly above to record the entire session. To commence the session, the animal was placed on the far side of the bright compartment, facing away from the rest of the box. A 5-minute period was allowed for the animal to explore and locate the dark. If the animal failed to locate the dark compartment, they were placed in the compartment for 1 min. During the training phase, the box was devoid of all partitions (i.e., obstacles). The partition was placed into the box for the testing phase.

***Elevated Plus Maze*** The principle of this assessment evaluates the conflict created between the animal’s exploratory drive and innate fear of open, elevated, and exposed areas (Vachon, 2013). The maze was placed on a marked area on the experimental room floor with the GoPro positioned directly above. It consisted of two open arms (50 x 10 cm) and two enclosed arms (50 x 10 x 40 cm), connected by a centre square (10 x 10 cm). The two open arms were opposite each other, and the maze was elevated to a height of 50 cm. To commence the test, the animal was placed in the centre of the maze facing a closed arm (Aguggia et al., 2013). The animal then had 5 min to freely explore the maze while behaviour was recorded.

**Euthanasia** Animals were euthanized via decapitation using a sharp guillotine. Euthanasia commenced at 7:00. Immediately following decapitation, the trunk blood was collected in EDTA tubes and placed on ice for the duration of the euthanasia. Various visceral organs were collected, including the heart, lungs, kidney, and liver. The brain was dissected and several brain parts were removed, including the prefrontal cortex, hypothalamus, hippocampus, and amygdala. Trunk blood was centrifuged at 1500 g for 15 min at 4 °C. Once all plasma was collected and labelled, it was placed in the biofreezer and stored at −80 °C until analysis. The remainders of the blood components were appropriately disposed of.

**Neurochemical Marker Analysis** Neurochemical analysis was performed on peripheral plasma corticosterone (CORT) and oxytocin (OT), as well as hypothalamic oxytocin (H-OT). All concentrations were evaluated using commercially available rat competitive ELISA kits. A pre-experiment was performed prior to each ELISA analysis to identify the optimal sample dilution to use for the respective experiments (CORT samples − 1:99; OT − 1:1000 [M] / 1:500 [F]; H-OT - undiluted). The ELISA manufacturers’ instructions were followed to complete all assays.

**Statistical Analysis** All statistical analysis was performed on GraphPad Prism, version 8.4.3. All behavioural videos were analysed using BORIS, Version 7.13. Normality and Gaussian distribution were determined using the Shapiro-Wilk normality test. Data from the electronic von Frey, acetone evaporation, and Hargreaves tests was analysed using repeated measures ANOVA followed by Bonferroni post-hoc test. Analysis of data from the EPM, LDB and biochemical assays were done using one ANOVA followed by Tukey’s post-hoc test. Finally, variables from the LDB and CORT assay where data was non-normally distributed was analysed using Kruskal-Wallis followed by Dunn’s multiple comparisons post-hoc test. All data were presented as means ± standard deviation (SD). Differences were considered significant if the p-value ≤ 0.05. Statistical significance is represented by the following system: * = p < 0.05; ** = p < 0.01; *** = p < 0.001; **** p < 0000.1.

## Results

This statement aims to provide a clear description of the results obtained in this study. During Phase 1, Wistar rats were exposed to *Paclitaxel*. Mechanical and thermal sensitivity was measured and compared at baseline and following *Paclitaxel* injection for each animal. ‘Baseline’ is defined as analgesic measures prior to receiving *Paclitaxel*/saline injections, while ‘post-injection’ refers to measures occurring after all injections were administered. Comparisons were also made between the two groups, denoted saline (n = 30) and *Paclitaxel* (n = 31). Phase 2 consisted of a 14-day treatment period, where comparisons were made between and within the following treatment groups over the intervention period: control (n = 10), *Paclitaxel* (n = 10), duloxetine (n = 10), oxytocin (n = 10), *Paclitaxel*-duloxetine (n = 10), and *Paclitaxel*-oxytocin (n = 11).

All animals remained healthy throughout the intervention, with no evidence of alopecia or diarrhoea. No significant weight loss was observed.

**Phase 1**

## Effect of Paclitaxel administration on mechanical hypersensitivity

**Fig. 1:**
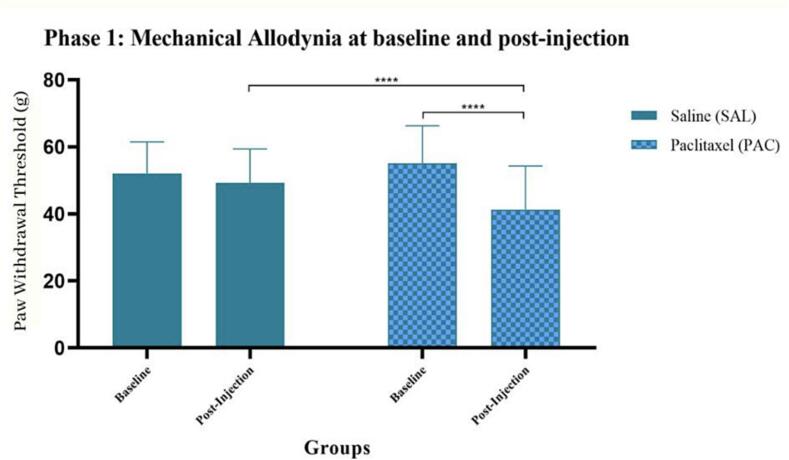
**Effect of Paclitaxel administration on mechanical hypersensitivity** Mechanical allodynia during Phase 1 in the following groups: saline (SAL, n = 30, F = 15) and Paclitaxel (PAC, n = 31, F = 16). Data are presented as mean ± SD. The group which received Paclitaxel exhibited a significant increase in mechanical sensitivity from baseline to post-injection recordings ****(PAC-BL vs PAV-PI), p < 0.0001. An overall Paclitaxel-effect (F (1, 58) = 20.48, p < 0.0001) was also identified between groups.

Mechanical allodynia was assessed in Wistar rats using the electronic von Frey filament. The paw withdrawal latency (PWL) was used as an indication of mechanical sensitivity. There was an overall significance when comparing baseline (BL) to post-injection (PI) measures within groups (F (1, 58) = 2066, p < 0.0001). The group which received Paclitaxel exhibited a significant increase in mechanical sensitivity from baseline to post-injection recordings ****(PAC-BL vs PAV-PI), p < 0.0001. An overall Paclitaxel-effect (F (1, 58) = 20.48, p < 0.0001) was also identified between groups. No significant difference was identified between group baseline measures. A comparison between groups’ post-injection measures exhibited a significant difference, with the Paclitaxel group demonstrating a reduction in paw withdrawal threshold (PWT), *(SAL-PI vs PAC-PI), p = 0.0123 (Fig. 1).

## Effect of Paclitaxel administration on thermal sensitivity

**Fig. 2:**
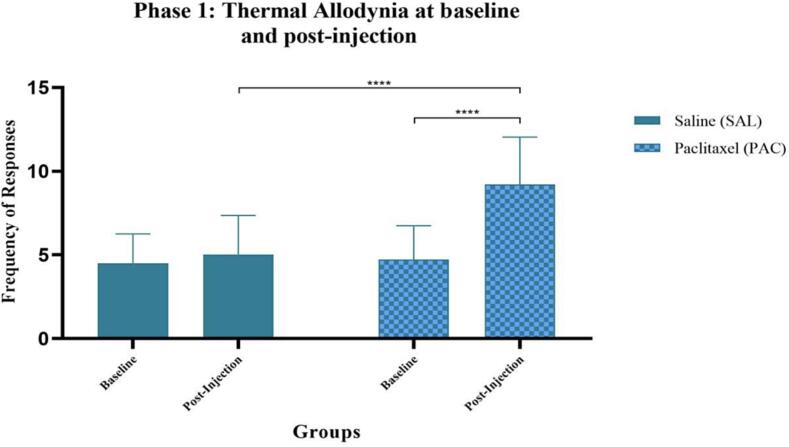
**Effect of Paclitaxel administration on thermal allodynia (cold sensitivity)** Thermal allodynia during Phase 1 in the following groups: saline (SAL, n = 30, F = 15) and Paclitaxel (PAC, n = 31, F = 16). The group which received Paclitaxel exhibited a significant increase in thermal sensitivity to a cold stimulus from baseline to post-injection recordings, ****(PAC-BL vs PAV-PI), p < 0.0001. An overall Paclitaxel-effect (F (1, 58) = 25.88, p < 0.0001) was also identified between groups.

Thermal allodynia was assessed in Wistar rats by exposing animals to a cold stimulus. The total frequency of responses was used as an indication of thermal sensitivity. There was an overall significance when comparing baseline (BL) to post-injection (PI) results within groups (F (1, 58) = 40.03, p < 0.0001). The group which received Paclitaxel exhibited a significant increase in thermal sensitivity from baseline to post-injection recordings, ****(PAC-BL vs PAV-PI), p < 0.0001. An overall Paclitaxel-effect (F (1, 58) = 25.88, p < 0.0001) was also identified between groups. No significant difference was identified between group baseline measures. A comparison between groups’ post-injection measures exhibited a significant difference, with the Paclitaxel group demonstrating an increase in thermal sensitivity, ****(SAL-PI vs PAC-PI), p < 0.0001 (Fig. 2).

**Fig. 3:**
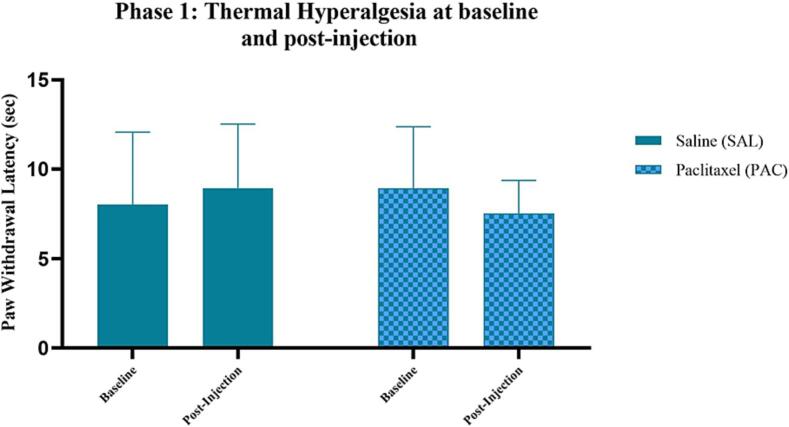
**Effect of Paclitaxel administration on thermal hyperalgesia (heat hypersensitivity)** Thermal hyperalgesia during Phase 1 in the following groups: saline (SAL, n = 30, F = 15) and Paclitaxel (PAC, n = 31, F = 16). Data are presented as mean ± SD. No significance was found between or within groups. No significance was found between or within groups.

Thermal hyperalgesia was assessed in Wistar rats using a localised heat source. Paw withdrawal latency (PWL) was used as a measure of thermal hypersensitivity. The following groups were assessed: saline (SAL) & Paclitaxel (PAC). No significance was found between or within groups (Fig. 3).

**Phase 2**

## Effect of treatment and intervention duration on mechanical sensitivity

**Fig. 4:**
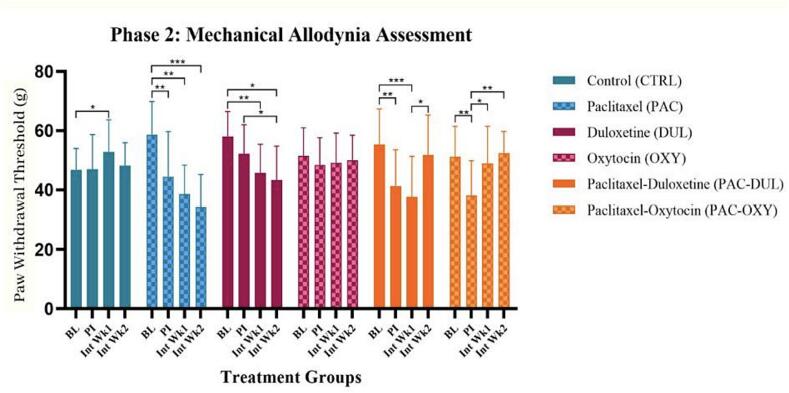
**Effect of treatment and intervention duration on mechanical sensitivity** Mechanical allodynia during Phase 2 in the following groups: control (CTRL, n = 10, F = 5), Paclitaxel (PAC, n = 10, F = 5), duloxetine (DUL, n = 10, F = 5), oxytocin (OXY, n = 10, F = 5), Paclitaxel-duloxetine (PAC-DUL, n = 10, F = 5), and Paclitaxel-oxytocin (PAC-OXY, n = 11, F = 6). A Paclitaxel-effect was observed in the animals that were administered Paclitaxel and treated with saline, indicated in the continuous increase in mechanical sensitivity over the time periods, **(PAC-BL) vs (PAC-PI), p = 0.0061; **(PACBL) vs (PAC-Int Wk1), p = 0.0016; ***(PAC-BL) vs (PAC-Int Wk2), p = 0.0002. Duloxetine did not have an effect during the first week of intervention. There was a duloxetine-effect found in the second week of intervention within the animals administered Paclitaxel and treated with duloxetine, as observed in the significant decrease in mechanical sensitivity, *(PAC-DUL-Int Wk1) vs (PAC-DUL-Int Wk 2), p = 0.0120. There was an overall oxytocin-effect observed, as the animals administered Paclitaxel and treated with oxytocin exhibited a significant decrease in mechanical sensitivity, *(PAC-OXY-PI) vs (PAC-OXY-Int Wk1), p = 0.0135; and intervention week 2 *(PAC-OXY-PI) vs (PAC-OXYInt Wk2), p = 0.0344 BL - baseline; PI - post-injection; Int Wk1 - Intervention Week 1; Int Wk2 - Intervention Week 2.

The effect of long-term treatment on mechanical allodynia was assessed using an electronic von Frey filament on Wistar rats. Repeated measures were taken during week 1 and 2 of intervention. There was an overall significance between time points (F (2.628, 141.9) = 16.97, p < 0.0001), as well as an overall interaction-effect between time and treatment (F (15, 162) = 6.895, p < 0.0001) (Fig. 3). A Paclitaxel-effect was observed in the animals that were administered Paclitaxel and treated with saline, indicated in the continuous increase in mechanical sensitivity over the time periods, **(PAC-BL) vs (PAC-PI), p = 0.0061; **(PACBL) vs (PAC-Int Wk1), p = 0.0016; ***(PAC-BL) vs (PAC-Int Wk2), p = 0.0002. Duloxetine did not have an effect during the first week of intervention. There was a duloxetine-effect found in the second week of intervention within the animals administered Paclitaxel and treated with duloxetine, as observed in the significant decrease in mechanical sensitivity, *(PAC-DUL-Int Wk1) vs (PAC-DUL-Int Wk 2), p = 0.0120. There was an overall oxytocin-effect observed, as the animals administered Paclitaxel and treated with oxytocin exhibited a significant decrease in mechanical sensitivity, *(PAC-OXY-PI) vs (PAC-OXY-Int Wk1), p = 0.0135; and intervention week 2 *(PAC-OXY-PI) vs (PAC-OXY Int Wk2), p = 0.0344 (Fig. 4).

## Effect of treatment and intervention duration on thermal sensitivity

*Acetone test*

**Fig. 5:**
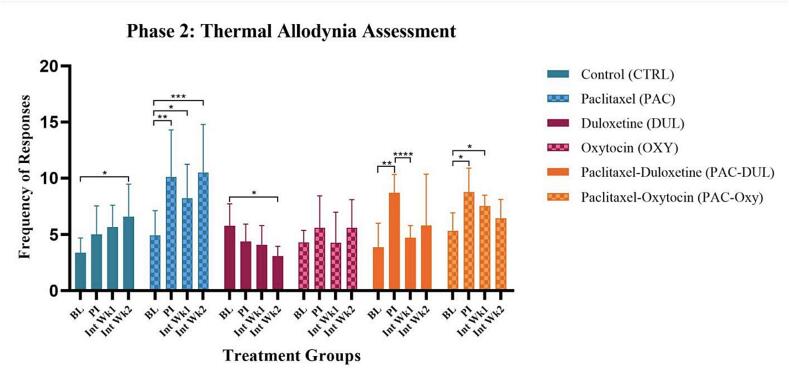
**Effect of treatment and intervention duration on thermal allodynia (cold sensitivity)** Thermal allodynia during Phase 2 in the following groups: control (CTRL, n = 10, F = 5), Paclitaxel (PAC, n = 10, F = 5), duloxetine (DUL, n = 10, F = 5), oxytocin (OXY, n = 10, F = 5), Paclitaxel-duloxetine (PAC-DUL, n = 10, F = 5), and Paclitaxel-oxytocin (PAC-OXY, n = 11, F = 6). The group administered Paclitaxel and treated with saline demonstrated an increase in thermal sensitivity in response to a cold stimulus throughout the intervention period, **(PAC-BL) vs (PAC-PI), p = 0.0261; *(PAC-BL) vs *(PAC-Int Wk1), p = 0.0499; ***(PAC-BL) vs (PAC-Int Wk2), p = 0.0220. A duloxetine-effect was observed within the group administered Paclitaxel, identified between post-injection and intervention week 1 measures, ****(PAC-DUL-PI) vs (PAC-DUL-Int Wk1), p < 0.0001. BL - baseline; PI - post-injection; Int Wk1 - Intervention Week 1; Int Wk2 - Intervention Week 2.

The effect of long-term treatment on thermal allodynia was assessed using the acetone test, where the total frequency of responses indicated thermal sensitivity. Repeated measures were taken during week 1 and 2 of intervention. A significant treatment-effect was found (F (5, 54) = 8.480, p < 0.0001), as well as a significant interaction-effect between time and treatment (F (15, 162) = 4.699, p < 0.0001), and finally a significant time-effect (F (2.827, 152.7) = 15.65, p < 0.0001) across the intervention period. The group administered Paclitaxel and treated with saline demonstrated an increase in thermal sensitivity throughout the intervention period, **(PAC-BL) vs (PAC-PI), p = 0.0261; *(PAC-BL) vs *(PAC-Int Wk1), p = 0.0499; ***(PAC-BL) vs (PAC-Int Wk2), p = 0.0220. A duloxetine-effect was observed within the group administered Paclitaxel, identified between post-injection and intervention week 1 measures, ****(PAC-DUL-PI) vs (PAC-DUL-Int Wk1), p < 0.0001 (Fig. 5).

*Hargreaves test*

**Fig. 6:**
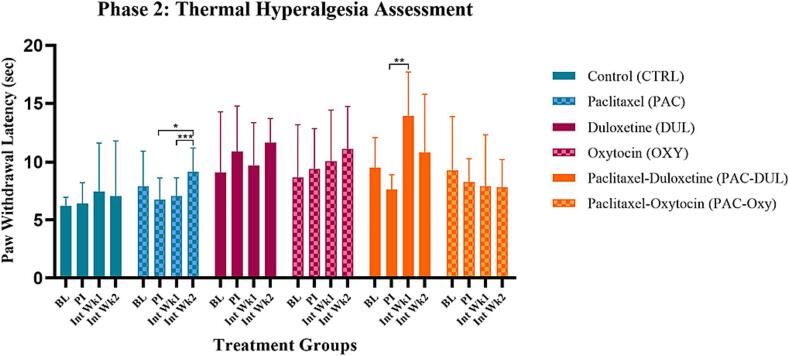
**Effect of treatment and intervention duration on thermal hyperalgesia (heat hypersensitivity)** Thermal hyperalgesia during Phase 2 in the following groups: control (CTRL, n = 10, F = 5), Paclitaxel (PAC, n = 10, F = 5), duloxetine (DUL, n = 10, F = 5), oxytocin (OXY, n = 10, F = 5), Paclitaxel-duloxetine (PAC-DUL, n = 10, F = 5), and Paclitaxel-oxytocin (PAC-OXY, n = 11, F = 6). The group administered Paclitaxel and treated with saline demonstrated an increase in thermal hypersensitivity throughout the intervention period. A significant overall treatment-effect was identified (F (5, 54) = 5.838, p = 0.0002). However, no significance was observed between specific groups. BL - baseline; PI - post-injection; Int Wk1 - Intervention Week 1; Int Wk2 - Intervention Week 2.

The effect of long-term treatment on thermal hyperalgesia was assessed using a localised heat source. Paw withdrawal latency (PWL) was used as a measure of thermal hyperalgesia. Repeated measures were taken during week 1 and 2 of intervention. A significant overall treatment-effect was identified (F (5, 54) = 5.838, p = 0.0002). However, no significance was observed between specific groups (Fig. 6).

## Anxiolytic Behavioural Assessment

### Effect of treatment and intervention duration on anxiety-like behaviour

Light dark box (LDB)

**Fig. 7:**
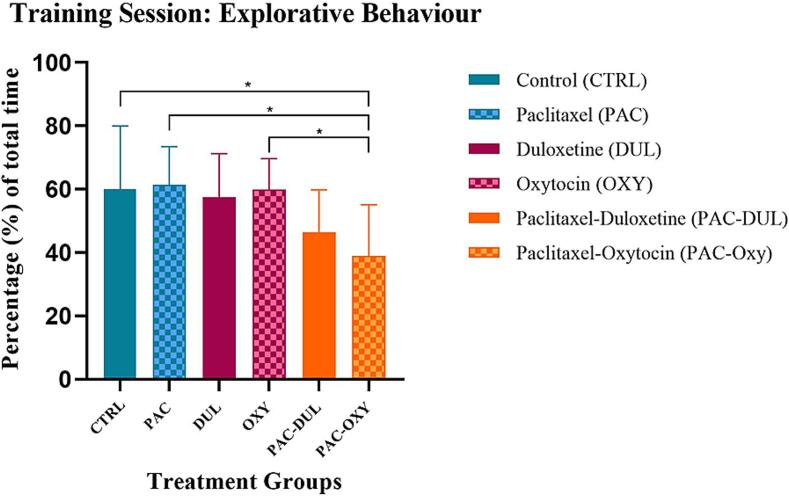
**Effect of treatment and intervention duration on anxiety-like behaviour during the light/dark box training session** Assessment of explorative behaviour in the light dark box training session in the following groups: control (CTRL, n = 10, F = 5), Paclitaxel (PAC, n = 10, F = 5), duloxetine (DUL, n = 10, F = 5), oxytocin (OXY, n = 10, F = 5), Paclitaxel-duloxetine (PAC-DUL, n = 10, F = 5), and Paclitaxel-oxytocin (PAC-OXY, n = 11, F = 6). Normality was confirmed using the Shapiro-Wilk test. Data are represented as mean ± SD. The neuropathic pain group receiving oxytocin as treatment demonstrated the least explorative behaviour compared to its counterparts, *p ≤ 0.05. The animals administered Paclitaxel and treated with oxytocin exhibited a decrease in time spent in the light compartment compared to respective counterparts, *(CTRL) vs (PAC-OXY), p = 0.0198; *(PAC) vs (PAC-OXY), p = 0.0106; *(OXY) vs (PAC-OXY), p = 0.0212.

Anxiety-like behaviour was assessed using the light dark box (LDB) post-intervention. Percentage of time spent in the light compartment is considered explorative behaviour, while percentage of time spent in the dark compartment is considered anxiety-like behaviour. A significant treatment-effect was found (F (5, 55) = 4.177, p = 0.0027) between the groups when observing the differences in percentage of time spent in the light compartment, specifically during the training sessions. The animals administered Paclitaxel and treated with oxytocin exhibited a decrease in time spent in the light compartment compared to respective counterparts, *(CTRL) vs (PAC-OXY), p = 0.0198; *(PAC) vs (PAC-OXY), p = 0.0106; *(OXY) vs (PAC-OXY), p = 0.0212. No other significance was found within the training and testing session (Fig. 7).

*Elevated Plus Maze (EPM)*.

**Fig. 8:**
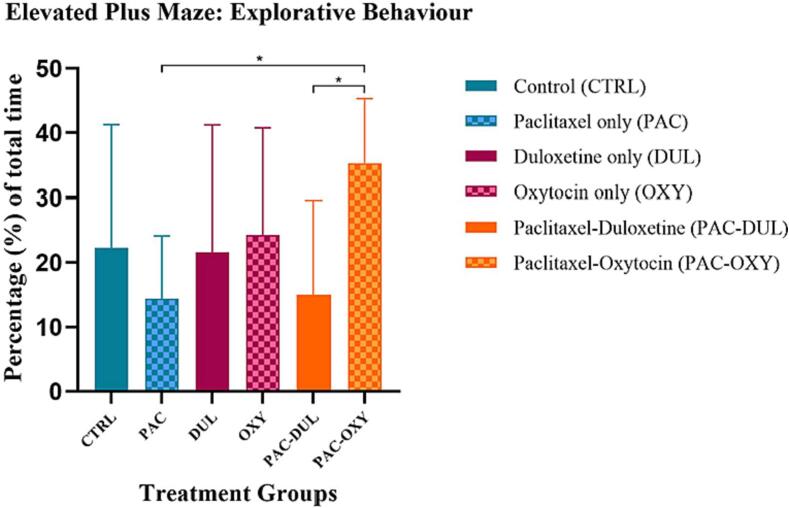
**Effect of treatment and intervention duration on explorative behaviour during the elevated plus maze** Explorative behaviour demonstrated in the elevated plus maze in the following groups: control (CTRL, n = 10, F = 5), Paclitaxel (PAC, n = 10, F = 5), duloxetine (DUL, n = 10, F = 5), oxytocin (OXY, n = 10, F = 5), Paclitaxel-duloxetine (PAC-DUL, n = 10, F = 5), and Paclitaxel-oxytocin (PAC-OXY, n = 11, F = 6). Data are represented as mean ± SD, *p≤0.05. There was an overall treatment-effect between groups (F (5, 55) = 2.593, p = 0.0355). There was no significance between the duloxetine, Paclitaxel, and control groups. An oxytocin-effect was found, as the animals administered Paclitaxel and treated with oxytocin displayed significantly more time in the open arm, indicative of explorative behaviour, *(PAC) vs (PAC-OXY), p = 0.0355; *(PAC-DUL) vs (PAC-OXY), p = 0.0416.

Anxiety-like behaviour was assessed using the elevated plus maze (EPM) post-intervention. Percentage of time spent in the closed arm was used as a measure of anxiety-like behaviour, while the percentage of time spent on the open arm was considered an indication of explorative behaviour. There was an overall significant treatment-effect between groups (F (5, 55) = 2.593, p = 0.0355) (Fig. 8). There was no significance between the duloxetine, Paclitaxel, and control groups. An oxytocin-effect was found, as the animals administered Paclitaxel and treated with oxytocin displayed significantly more time in the open arm, indicative of explorative behaviour, *(PAC) vs (PAC-OXY), p = 0.0355; *(PAC-DUL) vs (PAC-OXY), p = 0.0416. No statistical significance was observed for the percentage of time spent in the closed arms.

## Neurochemical Assessment

### Effect of treatment and intervention duration on neurochemical concentrations

Corticosterone

**Fig. 9:**
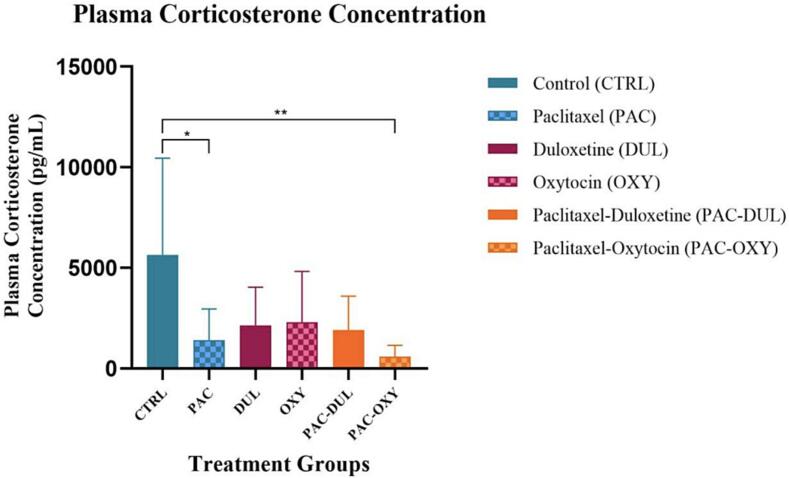
**Effect of treatment and intervention duration on plasma corticosterone concentrations** Peripheral plasma corticosterone concentration was measured in the following groups: control (CTRL, n = 10, F = 5), Paclitaxel (PAC, n = 10, F = 5), duloxetine (DUL, n = 10, F = 5), oxytocin (OXY, n = 10, F = 5), Paclitaxel-duloxetine (PAC-DUL, n = 10, F = 5), and Paclitaxel-oxytocin (PAC-OXY, n = 11, F = 6). A significant decrease in the corticosterone concentration within the circulation of the animals administered Paclitaxel and treated with oxytocin and saline was observed when compared to the control group; **(CTRL) vs (PAC-OXY), p = 0.0011, *(CTRL) vs (PAC), p = 0.0474.

Peripheral plasma corticosterone concentrations were assessed in Wistar rats. There was an overall treatment-effect observed (H (6, 61) = 17.59, p = 0.0035). A significant decrease in the corticosterone concentration within the circulation of the animals administered Paclitaxel and treated with oxytocin and saline was observed when compared to the control group; **(CTRL) vs (PAC-OXY), p = 0.0011, *(CTRL) vs (PAC), p = 0.0474 (Fig. 9).

Oxytocin

**Fig. 10:**
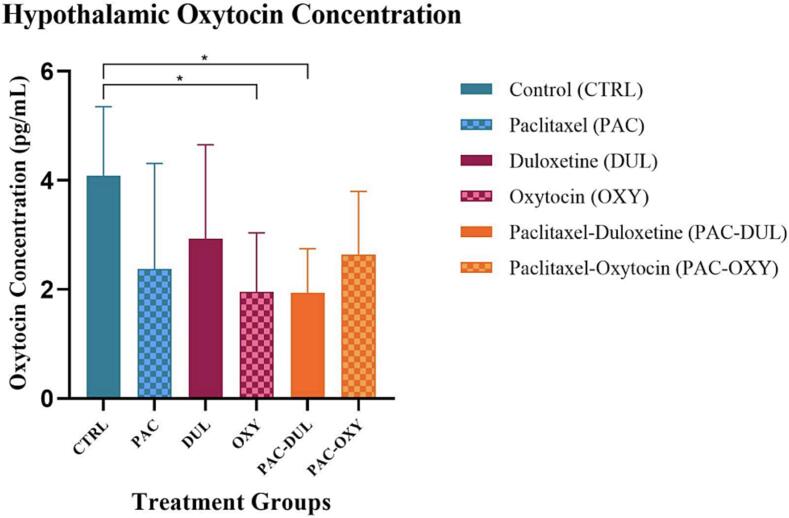
**Effect of treatment and intervention duration on hypothalamic oxytocin concentrations** Hypothalamic oxytocin concentration was measured in the following groups: control (CTRL, n = 10, F = 5), Paclitaxel (PAC, n = 10, F = 5), duloxetine (DUL, n = 10, F = 5), oxytocin (OXY, n = 10, F = 5), Paclitaxel-duloxetine (PAC-DUL, n = 10, F = 5), and Paclitaxel-oxytocin (PAC-OXY, n = 11, F = 6). The control group demonstrated a significantly increased hypothalamic oxytocin concentration compared to the oxytocin group, as well as the animals administered Paclitaxel and treated with duloxetine, respectively; *(CTRL) vs (OXY), p = 0.0327; *(CTRL) vs (PAC-DUL), p = 0.0186.

Hypothalamic oxytocin concentration was assessed in Wistar rats. A significant treatment-effect was observed (F (5, 47) = 2.972, p = 0.0206) (Fig. 10). The control group demonstrated a significantly increased hypothalamic oxytocin concentration compared to the oxytocin group, as well as the animals administered Paclitaxel and treated with duloxetine, respectively; *(CTRL) vs (OXY), p = 0.0327; *(CTRL) vs (PAC-DUL), p = 0.0186 (Fig. 10). Peripheral plasma oxytocin concentration was assessed in Wistar rats. No significance was identified between groups.

## Discussion

As the prevalence of cancer rises, a corresponding increase in chemotherapy utilisation is expected, subsequently resulting in an exponential rise in CIPN. Being a dose limiting condition, associated with severe impairments to one’s quality of life, the necessity to establish effective management of CIPN is vital. While duloxetine is considered the ‘gold standard’ pharmaceutical approach, it is associated with a plethora of adverse effects which escalates the overall burden to the patient. In order to identify alternative or augmentative therapeutic approaches, further research must be performed using the appropriate preclinical model. We therefore aimed to establish a model of CIPN in Wistar rats, using the chemotherapeutic drug Paclitaxel, and introduce an augmented long-term treatment alternative to the conventional gold standard pharmaceutical intervention. We introduced synthetic oxytocin administered intranasally as a novel treatment, to investigate the potential analgesic and anxiolytic effect it provided. We evaluated the changes in mechanical and thermal sensitivity over the intervention period as an indication of the analgesic potential of the various treatments.

Our findings showed that we were able to establish a model of chemotherapy-induced peripheral neuropathy pain, as the animals that received Paclitaxel exhibited a significantly greater mechanical and thermal sensitivity when compared to their control counterparts. It is well-established that CIPN affects both mechanical and thermal nociceptive responses (Omran, 2021), resulting in a hypersensitivity to these stimuli. Paclitaxel has been shown to induce morphological and electrophysiological alterations potentially responsible for axonal degeneration and demyelination, voltage-gated ion channel upregulation and receptor expression changes, impeding neuronal communication and resulting in the typical presentation associated with neuropathic pain (Omran et al., 2021, Staff et al., 2020). These mechanisms could contribute to diminished neuronal function and a reduction in fibre density responsible for epidermis innervations, ultimately contributing to the assemblage of behavioural changes observed in vivo. It is also suggested that these mechanisms facilitate other associated clinical symptom presentation of neuropathic pain, including numbness, tingling, ongoing pain, and allodynia and/or hyperalgesia (Griffiths, 2018).

We did not observe any significant changes in thermal hyperalgesia during Phase 1 of our study, findings which are in agreement with Griffiths (2018). Duggett (2016) found an upregulation of ROS within subpopulations of non-peptidergic C-fibers, responsible for mechanical stimuli processing only, in a *Paclitaxel*-induced peripheral neuropathy (PIPN) model, with no significant change in thermal-sensitive peptidergic fibers. It is possible that a higher dose of *Paclitaxel* is required to induce neurodegeneration extensive enough to cause underlying heat perception alterations (Griffiths, 2018). We also noted the substantial standard deviation within both groups at both time points. This may be a contributing factor to the lack of significance found. However, clinical trials have described CIPN symptoms as “numbness, tingling, shooting or stabbing pain, burning pain, cramping, and hypersensitivity to cold temperatures” (Staff, 2020). Therefore, the lack of heat sensitivity would align with these clinical presentations.

To further elaborate on the progression of the CIPN model, our findings from Phase 2 showed an exacerbation of CIPN-related symptoms without administered treatment, represented by the progressive sensitivity of nociceptive responses over the intervention period. This is suggestive of the importance of timely condition management. We observed peak mechanical and thermal sensitivity around Day 28 following initial injection, findings which parallels previous literature (Duggett, et al., 2016, Griffiths et al., 2018). One of the hallmark characteristics of chronic pain is central sensitization, caused by abnormal nociceptive hyperexcitability of the central nervous system, resulting in the presentation of progressive mechanical sensitivity over time (Yan, 2017). Further supporting the establishment of a CIPN model, our findings elucidate to a heightened presence of anxiety-like behaviour without treatment, a symptom often associated with chronic neuropathic pain (Toma, 2017), as demonstrated by a decreased preference for open arms in the elevated plus maze (EPM). CIPN is a disease which becomes exacerbated over time, as the central nervous system becomes increasingly involved, resulting in the increase in risk of developing severe affective disorders, such as anxiety, depression, anhedonia, and insomnia (Liu & Chen, 2014). Plausible mechanisms are associated with an upregulation of numerous inflammatory pathways in the DRG (Li, Mai and Liu, 2014) and pathological alterations in pain processing areas within the brain, including the amygdala (Liu & Chen, 2014; Wu *et al*., 2022), anterior cingulate cortex (Liu & Chen, 2014), and prefrontal cortex (PFC) (Roch, 2022). This increases neuronal excitability via channel activation, facilitating an increase in nociception and anxiety-like behaviour (Roch, 2022).

We did not find evidence of hyperactive hypothalamic–pituitary–adrenal (HPA) axis activity, as the corticosterone (CORT) concentration in the circulation was significantly lower when compared to the control group. Our CORT concentrations were well-below what is considered baseline based on previous literature. Interestingly, Kozachik and Page (2016) found an inverse correlation between plasma CORT levels and mechanical hypersensitivity. This may explain why we observed low plasma CORT concentrations with a reduced ability to tolerate mechanical stimuli.

There was a delayed analgesic onset for mechanical sensitivity in animals treated with duloxetine, with animals only exhibiting a reduction in sensitivity in the final intervention week. Chahal et al. (2020) observed a gradual increase in mechanical nociceptive responses in PIPN until 14 days after intervention had commenced. Thereafter, 30 mg/kg of daily duloxetine administered via oral gavage induced a significant antinociceptive effect. The delay observed in the attenuating effects of duloxetine on mechanical allodynia may be the result of when the intervention was introduced. Previous studies have demonstrated more immediate analgesic effects of duloxetine when treatment commenced prior or adjacent to Paclitaxel administration (Kato et al., 2021, Staurengo-Ferrari et al., 2022). Therefore, a longer intervention duration is needed to expand on these hypotheses. Interestingly, duloxetine did not offer an anxiolytic benefit, as demonstrated by the lack of inclination to spend time in the open arms of the EPM compared to respective counterparts. Staurengo-Ferrari (2022) identified that duloxetine’s efficacy was greater for oxaliplatin-induced neuropathy as opposed to PIPN, indicative of the importance of specificity regarding treatment. While duloxetine acts as a serotonin and norepinephrine uptake inhibitor (Rodrigues-Amorim, 2020), the predominant mechanism of action in a PIPN involves upregulated glutamatergic and inhibited GABAergic pathways (Omran, 2021). Therefore, there is a need to augment this intervention to enhance efficacy.

We found that oxytocin provided significant nociceptive response attenuation, as exhibited in the reduction of both mechanical and cold sensitivity during the intervention period. Our findings parallel those of González-Hernández (2019), who demonstrated the analgesic effects of repeated administration of exogenous oxytocin in a Wistar rat model of spinal nerve ligation. We deduce that the analgesic mechanisms of the aforementioned results can be explained by oxytocin’s potential to act at a central, spinal, and peripheral level. Oxytocin has an affinity to non-peptidergic C-fibers in the DRG (Boll, 2018), and a direct inhibitory effect on electrophysiological mechanisms (Duggett, 2016), making these plausible mechanisms providing attenuation of nociception and significant analgesic benefits in a CIPN model. Furthermore, oxytocin projections have been located on various pain processing regions, including the cingulate and insular cortices, PFC, thalamus (Boll, 2018), amygdala (Li, 2017), and PAG (Rash, Aguirre-Camacho and Campbell, 2014). Therefore, it is possible that oxytocin exerts potential central ameliorative effects via these projections, if centrally administered.

Oxytocin further demonstrated anxiolytic potential, observed in the preference to open arm activity, supplementing the analgesic behavioural results exhibited. Few studies have addressed the need for alternative interventions for emotional disorders associated with CIPN (Roch, 2022). Oxytocin is well-known for its anxiolytic properties, potentially mediated through action on brain areas including the amygdala, the anterior cingulate cortex, and areas of the PFC (Boll, 2018). Previous literature observed an attenuation of nociceptive and anxiety-like behaviour in a neuropathic model of common peroneal nerve (CPN) ligation following microinjections of oxytocin into the anterior cingulate cortex (ACC). Oxytocin reduced pre-long-term-potentiation (LTP) in the ACC of both male and female mice as well as mediated inhibitory pathway activity (Li, *et al.* 2021). Furthermore, evidence has suggested oxytocin indirectly facilitates excitation in the GABAergic system, mediated by PVN hypothalamic projections, offering an indirect analgesic effect (Xin et al., 2017). As PIPN’s mechanism has been associated with upregulated glutamatergic and inhibited GABAergic stimulation within the CNS (Omran, 2021), this may offer an explanation for the anxiolytic results observed. Interestingly, we did not observe consistent findings across the anxiety-like behavioural tests. Our findings are not consistent with previous literature that has found an increase in anxiety-like behaviour in models of neuropathic pain using the LDB protocol (Sieberg et al., 2018, Toma et al., 2017, Warncke et al., 2021). However, there is limited research supporting the use of the LDB in this particular model of CIPN. Warncke (2021) only identified an increase in anxiety-like behaviour in females receiving a high dose of Oxaliplatin (30 mg/kg cumulative dose), suggesting that the chemotherapy agent and dosage used must be considered when selecting this specific test. In support of this, Currie (2019) identified the importance of specificity regarding anxiolytic testing in a *meta*-analysis of CIPN in animal models. To obtain clarity regarding these results, the experimental procedure would need to be repeated with consideration taken regarding the potential confounding variables.

We further sought to validate our behavioural observations with neurochemical measures. Oxytocin induced ameliorative effects on circulating corticosterone within a CIPN model, as demonstrated by the significantly lower corticosterone concentration as compared to the control counterpart. As oxytocin is a well-known antagonist of the HPA axis (Tracy, 2015), we speculate the above-mentioned results can be explained through these mechanisms. While there is limited research on the effects of oxytocin in a chronic pain model, studies have demonstrated the attenuating potential of oxytocin in chronic stress models. Our findings are in agreement with Maikoo et al. (2022) who found intranasal oxytocin administration reduced circulating CORT levels in a febrile seizure model. Furthermore, Windle (2006) found centrally administered oxytocin significantly attenuated the plasma corticosterone concentration in an auditory stress animal model. As oxytocin receptors have been identified on numerous brain regions and ascending/descending pathways associated with chronic pain development and progression (Boll, 2018), we speculate that centrally administered oxytocin may have exerted its ameliorative effects by regulating activity in affected brain regions.

## Limitations and future studies

As this was a preliminary study, we did not introduce the intervention until after CIPN had been verified. Recent literature has demonstrated the importance of when intervention, in particular duloxetine, commences in relation to chemotherapy administration (di Cesare Mannelli, et al., 2017, Zhang et al., 2018; Lu *et al*., 2020; Staurengo-Ferrari, 2022). In addition, the time allocated to this study did not allow for further investigation into the optimal dosage needed for treatment. Further research will need to be conducted to establish a dose–response relationship in a Paclitaxel-induced model of neuropathic pain, as Han and Yu, (2009) found a dose-dependent relationship between centrally administered oxytocin and hind paw withdrawal latency (HWL). We also did not include a treatment group treated with a combination of oxytocin and duloxetine. An evaluation of the synergistic effect of these treatments in a model of CIPN would be the ideal progression for future studies. Furthermore, sex is known to be an under-research yet fundamental variable in chronic neuropathic pain models (Currie, 2019). While our model did include both males and females, we did not identify considerable differences between them. We could speculate on the reason for lack of heterogeneity; however, we did not include an analysis of gonadal hormones nor the oestrus cycle. Furthermore, it has been suggested that the anxiolytic test selected is model-specific, therefore a variety of affective disorder tests should be evaluated in this specific model, to ensure methodology optimization (Currie, 2019). Regarding methodology, the timing of sacrifice is important to note, as the animal’s levels would have been at their lowest in the morning. As blood collection was once-off, we were only provided with a momentary snapshot of these levels. In addition, research into ongoing pain-like behavioural assessment has been identified as an important outcome measure, as Griffiths (2018) have shown impairment of innate burrowing and wheel running behaviours occurring in parallel with the peak of evoked pain-like behaviours. The neuronal mechanisms involved in Paclitaxel-induced sensitivity likely differ between rodents and humans (Staff, 2020), and therefore it may be beneficial to extend research to human cells models to identify the effects of oxytocin in the presence of Paclitaxel cytotoxicity. It must also be considered that clinical symptoms are not absolute reflections of peripheral mechanisms (Omran, 2021). Therefore, the lack of subjective, electrophysiological, morphological, or central measures in a preclinical model could limit our comprehensive understanding of the interactive mechanisms involved in CIPN. While we measured the oxytocin concentration levels within the hypothalamus, we did not include an analysis of the dorsal root ganglion, a structure intimately involved in the development of chronic neuropathic pain (Duggett, et al., 2016, Staff et al., 2020, Yan et al., 2017).

## Conclusion

Chemotherapy-induced peripheral neuropathy (CIPN) remains to be a debilitating dose limiting side effect for patients fighting against cancer. As duloxetine is currently the only recommended treatment for CIPN management, the need for alternative or augmentative treatment modalities is imperative. Therefore, we sought to investigate the augmentative potential of the neuropeptide oxytocin in a model of CIPN. Our results support the hypothesis that intranasally administered oxytocin may augment the analgesic and anxiolytic effects of duloxetine in a chemotherapy-induced peripheral neuropathy model in a Wistar rat. Duloxetine demonstrated a greater inclination to attenuate thermal hypersensitivity as opposed to mechanical. It is plausible to postulate that oxytocin may offer the complementary mechanisms associated with mechanical sensitivity. Administered in conjunction, oxytocin and duloxetine may provide enhanced therapeutic effects in the treatment of CIPN. With more research, it may become evident of the potential role oxytocin may play in cancer- and chemotherapy-management.

## CRediT authorship contribution statement

**Michaela de Kock:** Methodology, Investigation, Resources, Writing – original draft, Visualization. **Sean Chetty:** Conceptualization, Validation. **Ahmed Sherif Isa:** Data curation, Formal analysis, Investigation, Methodology, Resources, Software, Supervision, Validation, Writing – review & editing. **Lihle Qulu-Appiah:** Conceptualization, Formal analysis, Funding acquisition, Methodology, Resources, Supervision, Validation, Visualization, Writing – review & editing.

## Declaration of competing interest

The authors declare that they have no known competing financial interests or personal relationships that could have appeared to influence the work reported in this paper.

## Data Availability

Data will be made available on request.
